# An assessment of technology-based service encounters & network security on the e-health care systems of medical centers in Taiwan

**DOI:** 10.1186/1472-6963-8-87

**Published:** 2008-04-17

**Authors:** Hsin Hsin Chang, Ching Sheng Chang

**Affiliations:** 1 Dept. of Business Administration, National Cheng Kung University, 1 University Rd., Tainan City 701, Taiwan; 2 Department of International Trade & Business Administration, Wenzao Ursuline College of Languages, 900 Mintsu 1st Rd., Kaohsiung City 807, Taiwan

## Abstract

**Background:**

Enhancing service efficiency and quality has always been one of the most important factors to heighten competitiveness in the health care service industry. Thus, how to utilize information technology to reduce work load for staff and expeditiously improve work efficiency and healthcare service quality is presently the top priority for every healthcare institution. In this fast changing modern society, e-health care systems are currently the best possible way to achieve enhanced service efficiency and quality under the restraint of healthcare cost control. The electronic medical record system and the online appointment system are the core features in employing e-health care systems in the technology-based service encounters.

**Methods:**

This study implemented the Service Encounters Evaluation Model, the European Customer Satisfaction Index, the Attribute Model and the Overall Affect Model for model inference. A total of 700 copies of questionnaires from two authoritative southern Taiwan medical centers providing the electronic medical record system and the online appointment system service were distributed, among which 590 valid copies were retrieved with a response rate of 84.3%. We then used SPSS 11.0 and the Linear Structural Relationship Model (LISREL 8.54) to analyze and evaluate the data.

**Results:**

The findings are as follows: (1) Technology-based service encounters have a positive impact on service quality, but not patient satisfaction; (2) After experiencing technology-based service encounters, the cognition of the service quality has a positive effect on patient satisfaction; and (3) Network security contributes a positive moderating effect on service quality and patient satisfaction.

**Conclusion:**

It revealed that the impact of electronic workflow (online appointment system service) on service quality was greater than electronic facilities (electronic medical record systems) in technology-based service encounters. Convenience and credibility are the most important factors of service quality in technology-based service encounters that patients demand. Due to the openness of networks, patients worry that transaction information could be intercepted; also, the credibility of the hospital involved is even a bigger concern, as patients have a strong sense of distrust. Therefore, in the operation of technology-based service encounters, along with providing network security, it is essential to build an atmosphere of psychological trust.

## Background

In such a world influenced by the rapid development of the computerization trend, overall medical costs have currently surged to cover the expense of use and development of new equipment, technology and rapidly expanded service volume. Enhancing service efficiency and quality has always been one of the most important factors to heighten competitiveness in the health care service industry [1,2]. In this fast changing modern society, e-health care systems are currently the best possible way to achieve enhanced service efficiency and quality under the restraint of healthcare cost control [3]. The electronic medical record system and the online appointment system are the core features in employing e-health care systems [4].

The purpose of a medical record is to store a patient's medical history systematically, for the continuity of medical care. However, a paper-based record system is often incompletely documented or fragmented with unclear handwriting, thus losing its usefulness and original function [5]. With an EMRS (electronic medical records system), not only is record keeping time for doctors and other health care staff reduced, but patient waiting time formerly extended by handwriting is cut [6], and tracking time on historically related records is also reduced just by accessing the integrated central system through the computer [7].

This makes it possible to provide instant medical examination and treatment, especially in the cases of emergency and drug allergy [8], boosting up the cure rate and work efficiency, and consequently increasing the service capacity.

Yield management is an important topic in the service industry for its unique features: fluctuating demands, limitation of service capacity and inability to inventory which makes the coordination of supply and demand extremely difficult [9]. The establishment of an online appointment system service is the appropriate solution for this situation with immediate effects on overall hospital operation efficiency [10]. For instance, from the human resources aspect, it provides sufficient time for health care staff in the service department to utilize medical resources and adjust the work load; as for medical facility equipment, it can be appropriately accessed and maintained according to schedule, to prolong the lifespan and reduce breakdown times; as for operating efficiency, it regulates patient's appointment scheduling, reducing patient's waiting time [10,11]. It is the solution to answering the future's growing demands for health care that is currently experiencing a shortage of manpower (inability to increase workers proportionally) caused by the rising aging population [12]. It is also the solution to achieving cost efficiency by minimizing waste of medical resources while enhancing service quality to the patient, thus lifting patient satisfaction and heightening hospital competitiveness [13].

Both the electronic medical record system and the online appointment system deploy electronic information exchange and application technology through the Internet to perform e-Commerce business transactions. Hence, any network security flaw would cause tremendous damage to individuals, companies or banks, producing a backlash effect. While the advancement of technology could enhance customer satisfaction, network security is definitely a crucial issue [14].

As a majority of studies have examined technical aspects of constructing an electronic medical record system and an online appointment system, the balance have been aimed at understanding the current state of the systems. This study implemented the Service Encounters Evaluation Model [15], the European Customer Satisfaction Index [16], the Attribute Model and the Overall Affect Model [17] for model inference, followed by thorough examination and investigation of practical cases.

Based on the above statements, the objectives of this study can be defined as follows: (1) To build a new integration model of technology-based service encounters; (2) To investigate whether technology-based service encounters have a positive impact on service quality; (3) To investigate whether technology-based service encounters have a positive impact on patient satisfaction; (4) To examine whether after experiencing technology-based service encounters, the cognition of the service quality has a positive effect on patient satisfaction; and (5) To scrutinize whether network security contributes a moderating effect between service quality and patient satisfaction.

## Literature Review and Research Hypotheses

The following section discusses the literature review and proposes the hypotheses on the topic of "the correlations among technology-based service encounters, service quality, patient satisfaction and network security."

### Service encounters

The service encounter is defined as "the dyadic interaction (face-to-face) between a service receiver and service provider" [18]. It is the core component of marketing in the service industry and possesses an impact on the scopes of service diversity, quality control, and customer satisfaction. Scholar's definition of service encounter is "the process in which a customer directly interacts with a service over a period of time" which mainly refers to the interaction between a customer and a service delivery system [19]. In short, a service encounter is over the period of time in which interaction occurs between a customer and service providers [20]. Scholar developed a model through experimentation to probe into the factors that impact customer's satisfaction/evaluation during the service encounter [15]. They then added a new set of 3 Ps (Participants, Physical Evidence, and Process) to the original 4 Ps to create the 7Ps of service marketing mix [21]. These are the essences of service encounters, especially the first 2 Ps (Participants, Physical); they exhibit a more realistic service encounter experience to the customer. While scholars have been attempting to define and classify a service encounter in recent years, they have been abiding by this broader definition rather than being limited to face-to-face interaction [22].

### Technology-based service encounters

In recent years, some scholars have used the terms of technology-based service [23,24], technology-enabled service delivery [25] and e-service encounter [26] to describe the platform of technology-focused service encounters. Scholars pointed out that more and more companies are going in the direction of e-Commerce (electronic commerce) [27], utilizing Internet technology to deliver services such as retail online shopping, airline ticket reservation, tracking packages or merchandise delivery service [28]. This was one of the reasons this study used the term "technology-based service encounter".

One of the main functions that attract people to a hospital's website is the online appointment system. By investigating people's preferences in using online information systems and constantly updating and improving the website, this online system offers a precious reference for hospitals to develop related health care systems, raise the medical flow efficiency and enhance the service quality [29]. As a result, it prompts hospitals to achieve the goal and efficiency of enterprise operation and to economize the cost [30]. An electronic medical record is not only a record that indicates a patient's medical information, but the core of the medical health care system; therefore, an electronic medical record should be seen as electronic information about the health condition and medical care of a patient throughout his entire life [11]. The EMRS will replace the paper-based record system as the main source of medical information in clinical practice, administrative management, medical education, research and legal demands [7]. Therefore, this study selected two authoritative southern Taiwan medical centers which provide electronic medical record and online appointment systems services to investigate the correlations among technology-based service encounter, service quality, customer's satisfaction and network security.

### Service quality (technology-based)

Scholars developed an assessment tool-E-QUAL to evaluate the service quality of electronic commerce businesses [31]. E-QUAL was developed based on PZB's SERVQUAL model; the empirical research on nine purely online travel agencies and fourteen hybrid travel agencies plus related literature regarding factors for website evaluation. It includes seven aspects: contents and purposes, approachability, guidance, design and display, response, background, personalization and customization. Based on Retailing Service Quality [32], scholars divided the service quality of online shopping into five aspects: entity's image, reliability, interpersonal interaction, problem solving and policy [33]. After interviewing consumers that were in the focus group from 4 banks and distributing 250 surveys to a convenient sample of electronic banking customers, scholars divided the service quality of electronic banking into eight aspects: accuracy, safety, approachability, convenience, bank's reliability, the ability to handle complaints, personalized demand and visual modality [34]. Based on PZB's SERVQUAL model, scholars developed a means to measure web-based service quality and divided it into seven aspects: visualization, reliability, response, assurance, empathy, quality of the information and communication integration [29,35].

### Patient satisfaction

Since scholars introduced the concept of customer satisfaction to marketing, customer satisfaction has been acknowledged by marketing literature as a central concept [36,37]. This only happens after a consumer has gone through the process of service and used the product. Satisfaction is an evaluation of emotional commitment [38]; the interpersonal interaction between a customer and service providers in retrospect [39]. And satisfaction with the interaction between the customer and company is a state of sentiment that will glide by as time passes [40]. Scholars investigated how product and service quality affects customer's transaction satisfaction [41,42]. The result demonstrated that the level of overall transaction satisfaction consumers have depends on their evaluation of service quality, quality of the product and the price [43]. From the above statement, we could conclude there is a positive correlation between service quality and patient (customer) satisfaction.

### Network security

Scholars believed as every industry was incessantly developing e-Commence on the Internet, the network security threats would gradually emerge [44,45]. There are 4 types of network security threats: interruption, causing system damage or paralysis; interception, caused by unauthorized users that steal useful information through the Internet (an unauthorized user can be a person, a program, or a computer); modification, alteration of the delivered data, causing unauthorized results and activity; and fabrication, the use of counterfeit identity to hack into the system.

Network security flaws are also a major threat to enterprises [14]. Scholars assumed that the safety and the privacy of networks will be settled as an important social issue besides the business process [46,47]. However, currently, there are only a few cases in U.S and Europe that utilize policy to protect privacy, and there is no evidence as yet, to prove that consumers are willing to spend money to protect their identities or their consuming behavior from being exposed in the market [11].

### Technology-based service encounters, service quality and patient satisfaction

Scholars inspected the consumers' preferences and evaluations of the self-service options of technology-based service encounters and discovered that different types of consumers hold different opinions and perceptions about the self-service options of technology-based service encounters [29,32]. There is a positive correlation between these different opinions and perceptions and consumers' inclination [13]. Thus he made an evaluation on the quality of self-service options by using the attribute model and the overall affect model. The attribute model concerned the customer's evaluation of the quality of technology-based self-service according to the characteristics of self-service options in the technology-based service encounter [48]. The overall affect model was an evaluation of the quality of technology-based self-service based on the customer's attitudes toward the technology-based service and the demands for interaction between the customer and service providers [49].

The ECSI (European Customer Satisfaction Index) model first associated customer satisfaction with the decisive factors, and then related it to the customer loyalty [16]. The decisive factors consisted of: a) company image; b) customer expectation; c) perceived value; d) perceived quality. Among them, the perceived quality was divided into two elements: the product (the tangible), which means the product quality was made up according to product attributes; the service (the intangible), which represents that the service factor was based on the interpersonal interaction with the customer, such as service providers' attitudes and the harmony of the service environment [50].

Scholar designed a Service Encounters Evaluation Model through experimentation to reveal the factors that influence customer's satisfaction during the process of service encounters [15]. Through the incorporation of the customer expectancy-disconfirmation model [37], the attribution theory [51], the service marketing mix [21] and the customer satisfaction [52], etc. in the service marketing field, he adopted a major theory of measuring the consumer's perception of the quality of service experienced, trying to give a more practical realization of the cause and effect that make influence on customer satisfaction during the process of service encounters. Scholars brought up the technology infusion matrix based on the 2 aspects of service encounters: the driving factors and the users [20]. It concluded that customers were able to use technology-based service encounters efficiently to enhance the experience, thus generating customer satisfaction. From the above statements, the following hypotheses are formed:

***Hypothesis 1: Technology-based service encounters have a positive impact on service quality***.

***Hypothesis 2: Technology-based service encounters have a positive impact on patient satisfaction***.

***Hypothesis 3: Service quality has a positive effect on patient satisfaction***.

### Network security, service quality and patient satisfaction

Scholars believed it is difficult to utilize one set of standard network security software to protect the entire enterprise, due to the complexity, diversity, and scattered structure of enterprise websites [44,47]. Constantly updating network security systems is essential to cope with the crafty attacks and vicious threats of computer viruses, worms, spyware and hackers [14]. Scholars held an opinion that the Internet will help the flow of enterprise information. Today with advanced Information Technology, multimedia data (including audio, video, and graphic data) makes resource communication and sharing more complicated [47,53]. Although advances in diverse technology raise the level of customer satisfaction, they also increase the maintenance cost of network security to prevent a backlash in customer satisfaction [11].

Scholars main research area was the information security risk control strategy [54]. Through tangible case studies, they discovered that the main threat to enterprise information security was internal employees. Scholars believed, due to the rapid growth of global commerce and enterprise websites, the network control of information storing, retrieving, user authentication and risk control have become the crucial points to protect enterprise network security from an internal or external attack [55,13]. To employers, these restraints have greatly affected the information service quality and user satisfaction [14]. From the above statements, network security (confidentiality, integrity, certification and non-repudiation) has become a moderator to service quality and patient satisfaction, thus hypothesis 4 is formed:

***Hypothesis 4: Network security contributes a positive moderating effect between service quality and patient satisfaction***.

## Methods

The structure of this study was based on the motive, purpose and literature reviews, and subsequently hypotheses were formed, a questionnaire was constructed and a sampling survey was conducted. The study also explained the composition of all the constructs and the method of data analysis, as follows.

### Research framework

**Figure 1:**
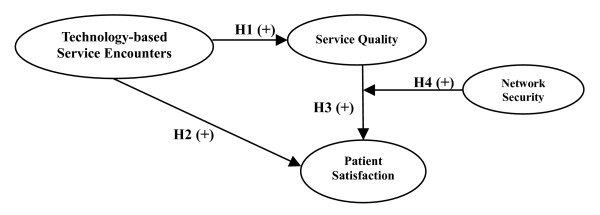
Conceptual framework of the relationship among technology-based service encounters, service quality, patient satisfaction and network security.

Technology-based service encounters are expected to enhance the efficiency and quality in the health care service industry and furthermore to raise service quality and patient satisfaction. As shown in figure 1, technology-based service encounters influence service quality and patient satisfaction, and service quality has an impact on patient satisfaction, while network security has a moderating effect between service quality and patient satisfaction. The framework of this study is shown in figure 1:

### Research hypotheses

This study aims to discuss the relationship among technology-based service encounters, service quality, patient satisfaction and network security, in which hypotheses are proposed according to research objectives, literature review, and research framework, as follows:

***Hypothesis 1: Technology-based service encounters have a positive impact on service quality***.

***Hypothesis 2: Technology-based service encounters have a positive impact on patient satisfaction***.

***Hypothesis 3: Service quality has a positive effect on patient satisfaction***.

***Hypothesis 4: Network security contributes a positive moderating effect between service quality and patient satisfaction***.

### Sampling Method

Two authoritative southern Taiwan medical centres which provide both electronic medical record system and the online appointment system services were selected for this study to examine the correlation among technology-based service encounters, service quality, patient satisfaction and network security by means of questionnaires. To understand the influence of online appointment and electronic medical record systems in technology-based service encounters on perceived service quality and patient satisfaction, as well as the moderating effect of network security on service quality and patient satisfaction, the chosen participants were patients who had experienced the electronic medical record system and the online appointment system services of technology-based service encounters. 700 questionnaires were distributed. Among the total return of 607 copies, 17 incomplete questionnaires were removed, with a valid return rate of 84.3%. The 590 valid questionnaires were used.

### Data Analysis Method

This questionnaire was constructed as a self-designed structural questionnaire after consulting related articles and case studies. It employed the 5-point Likert-type rating scale to collect data, after which 3 professors and 2 medical specialists had many discussions and revisions to evaluate its effectiveness. Finally, to effectively measure the correlation among technology-based service encounters, service quality, patient satisfaction and network security, 46 clinical patients were pre-tested to verify the importance, completeness and pertinence of the questions as well as to eliminate any ineffective questions. Because patients directly filled in the questionnaires in the independent variable and dependent variable sections, a single source bias (the deviation caused by the common method variance) might occur [56]. Thus, to avoid and reduce the occurrence of common method variance which might raise the possibility of overestimation and underestimation by the patients, we adopted: 1) a participant information confidentiality approach, using an anonymous method to reassure the participants; and 2) a concealed purpose approach, by not revealing the variables of every aspect in the questionnaire to reduce the doubts and suspicions that participants may have. After the questionnaires were assembled, we first inspected and sorted out the questionnaires by removing the incomplete ones while numbering and filing the valid questionnaires. We then used SPSS 11.0 and the Linear Structural Relationship Model (LISREL 8.54) to analyze and evaluate the data. This included:

1) Reliability analysis (see table 1): Cronbach's α value is generally between 0 and 1; however, the lowest acceptable value should be set as 0.7 [57]. All the question items had reached this standard.

**Table 1:** Factor Naming and Reliability Analysis of Constructs

**Constructs**	**Factor Naming**	**Cronbach's α (> .6)**
**Technology-based Service Encounters**		
	E-workflow (the Online Appointment System Service)	**0.832**
	E-facility (the Electronic Medical Record)	**0.912**
**Service Quality**		
	Reliability	**0.891**
	Credibility	**0.862**
	Customization	**0.811**
	Convenience	**0.912**
**Patient Satisfaction**		
	Patient Satisfaction	**0.816**
**Network Security**		
	Confidentiality	**0.802**
	Integrity	**0.815**
	Certification	**0.803**
	Non-repudiation	**0.862**

2) Constructs' discriminant validity (see table 2): To evaluate whether the factors of every construct (when 2 or more factors exist) possess discriminant validity, we calculated Chi-squares of every 2 factors in every construct under limited and unlimited conditions, and then we calculated the difference between these two values (χ^2^). If two factors' χ^2 ^are greater than 3.84 (χ^2^_1, 0.05 _> 3.84), discriminant validity exists between the two factors. The results showed the value of every χ^2 ^was greater than 3.84. This indicated every factor in the constructs was different, thus they all possessed discriminant validity [58,59].

**Table 2:** Discriminant Validity of Constructs

**Technology-based Service Encounters**			
Factors/	E-workflow (the Online Appointment System Service)		
E-facility (the Electronic Medical Record)	**31.07**		
**Service Quality**			
Factors/	Reliability	Credibility	Customization
Credibility	**171.13**		
Customization	**193.61**	**176.45**	
Convenience	**212.23**	**237.61**	**252.93**

**Network Security**			
Factors/	Confidentiality	Integrity	Certification
Integrity	**138.15**		
Certification	**65.24**	**78.17**	
Non-repudiation	**71.31**	**75.32**	**62.54**

***Note***. The Δχ^2 ^value between every two factors is > 3.84 in every construct.

3) Constructs' convergent validity (confirmatory factor analysis, see table 3): From the measurement model, we can derive the estimate parameter (λ) between each latent variable and manifest variable to know whether the estimate parameter (λ) is significant to evaluate the convergent validity. Thus, as in table 3, the values of t were all greater than 2, which demonstrated a fair degree of convergent validity. In addition, the values of composite reliability of all the constructs were all greater than 0.6, which showed the manifest variables were able to derive the latent variables. This meant the variables had high internal consistency [58,59].

**Table 3:** Convergent Validity of Constructs (Confirmatory Factor Analysis)

Constructs	Question Items	Standardized Factor Loading	Measurement Error	Composite Reliability
**Technology – based Service Encounters**	The hospital provides the online appointment system service.	0.756*	**0.428**	**0.82**
	The online appointment system service is very useful to me.	0.856*	**0.267**	
	The online appointment system service is easy for me to use.	0.769*	**0.409**	
	I can easily make an appointment through the online appointment system service.	0.831*	**0.309**	
	The hospital provides the electronic medical record system.	0.842*	**0.291**	
	I think we should use the electronic medical record system.	0.762*	**0.419**	
	Using the electronic medical record system is beneficial to me/patient.	0.773*	**0.402**	
	Overall, I think the electronic medical record system has a practical value to patients.	0.715*	**0.489**	

**Service Quality**	Information technology service (the online appointment system/electronic medical record system) can precisely record patients' medical care history.	0.853*	**0.272**	**0.83**
	The chance of information technology service (the online appointment system/electronic medical record system) making mistakes is slim.	0.841*	**0.293**	
	Information technology service (the online appointment system/electronic medical record system) can let patients finish the process of medical care.	0.786*	**0.382**	
	Information technology service (the online appointment system/electronic medical record system) can carry out the promise to patients.	0.850*	**0.278**	
	Information technology service (the online appointment system/electronic medical record system) has a good privacy protection system.	0.632*	**0.601**	
	Information technology service (the online appointment system/electronic medical record system) is trustworthy.	0.747*	**0.442**	
	I have confidence in information technology service (the online appointment system/electronic medical record system).	0.882*	**0.222**	
	Information technology service (the online appointment system/electronic medical record system) can satisfy my individual needs.	0.815*	**0.336**	
	Information technology service (the online appointment system/electronic medical record system) provides an extensive explanation about the use of the system.	0.822*	**0.324**	
	Information technology service (the online appointment system/electronic medical record system) can reduce the treatment time.	0.759*	**0.424**	
	Information technology service (the online appointment system/electronic medical record system) provides a rapid medication treatment result.	0.712*	**0.493**	
	Information technology service (the online appointment system/electronic medical record system) is very convenient.	0.837*	**0.299**	

**Patient Satisfaction**	Information technology service (the online appointment system/electronic medical record system) meets my expectations.	0.825*	**0.319**	**0.86**
	I am satisfied with information technology service (the online appointment system/electronic medical record system).	0.836*	**0.301**	
	I am a loyal customer of this hospital.	0.829*	**0.313**	
	I received very satisfactory service from the information technology service (the online appointment system/electronic medical record system).	0.811*	**0.342**	

**Network Security**	Information technology service (the online appointment system/electronic medical record system) protects personal privacy and prevents unauthorized use.	0.702*	**0.507**	**0.79**
	It is safe to provide personal information in information technology service (the online appointment system/electronic medical record system).	0.722*	**0.479**	
	I think information technology service (the online appointment system/electronic medical record system) is dependable.	0.863*	**0.255**	
	Information technology service (the online appointment system/electronic medical record system) protects information from being modified and distorted.	0.746*	**0.443**	
	I believe information technology service (the online appointment system/electronic medical record system) authenticates user's identity.	0.723*	**0.477**	
	I can trust the records provided in information technology service (the online appointment system/electronic medical record system).	0.798*	**0.363**	
	I feel comfortable leaving personal information in information technology service (the online appointment system/electronic medical record system).	0.733*	**0.463**	
	I think information technology service (the online appointment system/electronic medical record system) is honest.	0.785*	**0.384**	
	I think information technology service (the online appointment system/electronic medical record system) avoids patient denial of records.	0.796*	**0.366**	
	I believe information technology service (the online appointment system/electronic medical record system) is reliable.	0.865*	**0.252**	

***Note***. * t-value > 2.

4) Descriptive statistical analysis (see table 4): Characteristics of samples.

**Table 4:** Descriptive Statistics of Samples (N = 590)

Demographic Variables	*n*	(%)
**Hospitals**		
Medical Center A	**323**	**54.7**
Medical Center B	**267**	**45.3**
**Sex**		
Male	**312**	**52.9**
Female	**278**	**47.1**
**Age**		
20 or below	**61**	**10.3**
21–30	**200**	**33.9**
31–40	**141**	**23.9**
41–50	**84**	**14.2**
51–60	**45**	**7.6**
61–70	**31**	**5.3**
71 or above	**28**	**4.7**
**Marital Status**		
Not married	**249**	**42.2**
Married	**341**	**57.8**
**Educational Level**		
Elementary or below	**64**	**10.8**
Junior high school	**42**	**7.1**
Senior high (Vocational) school	**176**	**29.8**
College	**136**	**23.1**
University or above	**172**	**29.2**

**Figure 2:**
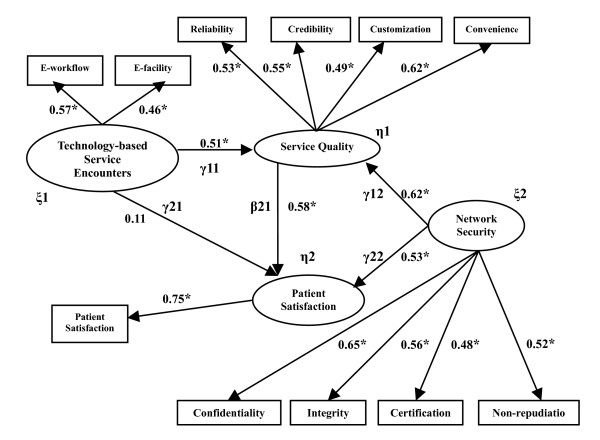
The Linear Structural Relationship Model of the correlation among technology-based service encounters, service quality, patient satisfaction, and network security.

5) Structural Equation Modeling (SEM) (see table 5 and figure 2).

**Table 5:** Overall Goodness of Fit

Fitness Statistics	χ^2^/df.	GFI	AGFI	NFI	RMSR
Standard Value	<3	>0.9	>0.8	>0.9	<0.08
**Model Statistic**	**2.78**	**0.92**	**0.89**	**0.90**	**0.045**

*Note*. χ^2^/df. = Ratio of Chi-square, GFI = Goodness of Fit Index, AGFI = Adjusted GFI, NFI = Normal Fit Index, RMSR = Root Mean Square of Standardized Residual.

## Results

### Characteristics of Samples

Most of the outpatients in the samples were male. In terms of age, the largest single group were 21~30 (33.9%), and the majority were between 20 and 50 years of age (72%). This demonstrated the fact that outpatients of the samples were not entirely middle-aged and elderly. In terms of educational level, most were college or senior high school educated (52.3% and 29.8%), which should be attributable to the general improvement in citizens' education, and most were married (57.8%) (see table 4).

### Structural Equation Modeling (SEM)

Using the Linear Structural Relationship Model to examine the relationship among technology-based service encounters, service quality, patient satisfaction and network security (see table 5 & figure 2).

### The Correlation among Technology-based Service Encounters, Service Quality, patient Satisfaction and Network Security

The objective of this study was to investigate the correlation among technology-based service encounters, service quality, patient satisfaction and network security utilizing the Linear Structural Relationship Model [58,59]. The results were according to the following statement (see table 5 and figure 2).

#### The correlation between technology-based service encounters and service quality

As shown in figure 2, from the correlated parameter estimates of technology-based service encounters and service quality, with a path coefficient of .51 > 0 (t = 12.61), it has achieved the level of significance (p < .05), and shown positive correlation. So, technology-based service encounters have a positive significant influence on service quality. Thus, ***Hypothesis 1 ***was confirmed.

#### The correlation between technology-based service encounters and patient satisfaction

As shown in figure 2, from the correlated parameter estimates of technology-based service encounters and patient satisfaction, with a path coefficient of .11 > 0 (t = 1.05). As it shows a positive correlation, but has not achieved the level of significance (p < .05), so technology-based service encounters do not have a positive significant influence on patient satisfaction. Thus, ***Hypothesis 2 ***was not confirmed.

#### The correlation between service quality and patient satisfaction

As shown in figure 2, from the correlated parameter estimates of service quality and patient satisfaction, with a path coefficient of .58 > 0 (t = 19.75), it has achieved the level of significance (p < .05), and shown positive correlation. So service quality has a positive significant influence on patient satisfaction. Thus, ***Hypothesis 3 ***was confirmed.

#### The correlation among network security, service quality and patient satisfaction

As shown in figure 2, from the correlated parameter estimates of network security to service quality and patient satisfaction, with path coefficients of .62 and .53 which are both greater than 0 (the values of t were 22.78 and 15.63), they have achieved the level of significance (p < .05), and shown positive correlation. So network security has a positive significant influence on service quality and patient satisfaction at the same time, which means network security has a positive moderating effect on service quality and patient satisfaction [58,59]. Thus, ***Hypothesis 4 ***was confirmed.

## Discussion

### Conclusion and implications

The objective of this study is to investigate the correlation among technology-based service encounters, service quality, patient satisfaction and network security. The academic and practical meanings are discussed in the following:

### Academic implications

1) Our findings support the statement that technology-based service encounters will positively influence service quality **(*Hypothesis 1*)**. This agrees with the assertions of previous relevant studies. For example, in the service convenience model from [60], consumers' cognition of convenience in technology-based service had a positive effect on their evaluation of service quality. Scholars pointed out that the banking industry actively sets up an electronic service procedure and delivery system to control costs, attract new clients and satisfy the expectations of convenience and innovative hi-tech from the customers [61]. The self-service options of the technology-based service system that the banking industry had set up could reduce the cost of personnel and improve the efficiency of operation [62,63]. It also had a positive effect of enhancing banking service quality.

2) Our findings also support the statement that service quality will positively influence patient satisfaction **(*Hypothesis 3*)**. This agrees with assertions made in previous studies, such as that by [37], which pointed out that service quality was the preceding variable to customer satisfaction. Therefore, good service quality would be sufficient to achieve customer satisfaction [39]. The research of scholars showed the quality of self-service options of a technology-based service system could enhance customer satisfaction [64]. Scholars addressed the topic from a supply and demand aspect [65]. From the supply side, a company could reduce staff to lower the cost of interpersonal service delivery by adopting self-service options in a technology-based service [65]. The innovation of technology-based service not only minimizes service providers' routine work load but could also satisfy the service demands from the consumer, enhance service quality and consequently raise the level of customer satisfaction [1].

3) The results of this study demonstrated technology-based service encounters to patient satisfaction **(*Hypothesis 2*) **do not have an obvious positive correlation relationship (the t-value of the path γ21 is not significant). Implication of this result confirms the studies of a number of researchers [15,66,67]. That is, the attributes of service encounters are in fact the same as a part of the service quality. Moreover, these service encounters attributes would usually directly affect service quality and consequently enhance patient satisfaction, but not directly affect patient satisfaction.

4) The results of this study showed that network security has a noticeablely positive influence on service quality and patient satisfaction **(*Hypothesis 4*)**. This means (1) network security has a positive effect on service quality; (2) network security has a positive effect on patient satisfaction; (3) network security has a positive moderating effect on service quality and patient satisfaction; and (4) because network security has a positive moderating effect on service quality and patient satisfaction, we should notice that there are two ways in which network security affects patient satisfaction: (a) direct effect: network security directly effects patient satisfaction; (b) indirect effect: service quality is an intermediate variable to affect patient satisfaction indirectly. Here are cases in which some past researchers' viewpoints corresponded to the above statement: scholars had built, from the customers' perspective, a service quality model, believing that one of the factors that affected service quality was safety [66]. Scholars found in their research that the only way to increase consumers' confidence and satisfaction in network technology service was to strengthen network security, which includes privacy protection and secure network transaction service [68]. In short, network security is the crucial factor in successfully promoting network technology-based or computer related services. Scholars discovered from their study that the reason users did not want to accept computer network technology-based service was due to having doubts on network security and lack of confidence in the service quality of technology-based services [24]. Thus, while network security enhances service quality and customer satisfaction, it also decreases service quality and customer satisfaction in some ways.

#### Practical implications

1) From the literature review we found, the Service Encounters Evaluation Model and the European Customer Satisfaction Index have rarely been applied to the topic of patients' satisfaction with technology-based service in health care service. However, from the results of this study, the model we built (for patients' satisfaction with technology-based service) showed a reasonable confirmation effect. This model can efficiently predict the cognition of service quality and patient satisfactions after patients (customers) have experienced the electronic medical record system (EMRS) and online appointment system service.

2) From the structural relationship model of technology-based service encounters, service quality, patient satisfaction and network security, it revealed that the impact of electronic workflow (online appointment system service) on service quality was greater than electronic facilities (EMRS) in technology-based service encounters. Scholar believed the chances of employing Internet technology in health care will increase as information technology continues to advance [69]. For example, the clinical, financial and customer data can more easily be stored, analyzed and delivered in the health care system and insurance organizations. Scholar believed the utilization of the Internet in the health care system includes education, continuing medical education, examination, consultation, support, clinical trials, diagnosis, analysis, interaction, cooperation, information flow, online support, online communication competition, electronic commerce, research, documentation and multimedia [70]. Therefore, convenience and credibility such as a good privacy protection system, fully carrying out the promise of information technology service to patients, are the important factors of service quality in technology-based service encounters that patients demand, as medical centers have employed far more sophisticated Internet technology in health care system (particularly in the EMRS and the online appointment system). They are also the factors that have the most effect on customer satisfaction. Enhancing service efficiency and quality has always been one of the most important factors to heighten competitiveness in the health care service industry. Service quality can be evaluated by customers' waiting time for service (convenience and response). In recent years, as the health care industry has become more intensely competitive, and consumers have been more conscious of their rights, enhancing service quality and patients' satisfaction have long been an important issue for every hospital. In the index of service quality, hospital waiting time (convenience) is the factor that most directly impacts patients and affects the patients' cognition of hospital service quality.

3) The result of this study also revealed that during the technology-based service encounters in the health care industry, credibility is one of the most important items in service quality. After all, privacy is still the most important thing that patients (customers) care about in the health care industry. Thus, while patients emphasize credibility most in service quality, network security also possesses a noticeable positive moderating effect on service quality and patient satisfaction in the entire Structural Relationship Model (Network security has an obvious positive effect on service quality and patient satisfaction. In other words, the better network security is, the more positive effect service quality has on patient satisfaction.) From a business transaction view of the privacy issue, privacy itself was not the problem; the problem was whether the use of personal information could be guaranteed or an agreement could be reached in technology-based service encounters. In the utilization of health care technology-based service encounters, due to the openness of networks, network security in business transactions has become an important topic; thus, we need to adopt the network security technology to ensure the safety of transactions in the utilization of technology-based service encounters. Because there is no face-to-face interaction in the network, consumers worry that transaction information could be intercepted; also, the credibility of the hospital involved is even a bigger concern, as consumers have a strong sense of distrust. Therefore, in the operation of technology-based service encounters, along with providing network security, it is essential to build consumers' psychological trust.

4) Hospitals should focus on how to enhance service quality to their patients because it has a positive correlation with the ratio of returning patients and hospitals' profit. Also, the influence of technology-based service encounters on service quality in health care cannot be ignored. The utilization of technology-based service encounters provides convenience and saves time, money and energy for organizations and patients/customers. From service providers' viewpoint, hospitals could learn how patients rate their organization's service quality through patients' cognition of technology-based service encounters, and further improve their service standard. Conversely, patients (customers) can evaluate the merits of the service provided from the patients' standpoint on technology-based service encounters to decide whether to continue using their service. Scholars pointed out: the key point whether consumers choose service with personal interaction or technology-based encounters (for example, withdrawing money from an ATM or a bank counter; an electronic medical record or a paper-based one; using an online appointment system or making an appointment at the scene) depends on whether technology-based service encounters can bring convenience and comfort in usage [64]. However, while consumers perceive the convenience of using technology-based service encounters, they may be less comfortable using it. They will likely avoid using technology-based service encounters. This will not only increase hospitals' costs, and reduce their efficiency and service quality, but also cause hospitals to lose a number of patients/customers.

## Research limitations

1. This study adopted the electronic medical record system and online appointment system service as an example of the utilization of technology-based service encounters. It doesn't mean that it is suitable for all technology-based service encounters. However, the structure and the model of this study provide a reasonable degree of validity and reliability.

2. The questionnaire can merely probe into the attitude participants hold toward the questions. To some degree, while the questions may lead to some subjective answers, it is difficult to derive participants' true opinions on the subject. Thus, we suggest future researchers conduct in-depth interviews with the participants and utilize quantitative and qualitative approaches to obtain a more definitive result.

3. This study selected only the patients that were from two authoritative southern Taiwan medical centers as the sample. Because it did not cover all the hospitals, the conclusions reached by this study may be somewhat limited. And it also used one particular time period only, which was unlikely to derive a thorough investigation on long term effective factors. We suggest using a multiple time period approach for follow-up research. By not being limited to a particular period of time, researchers can better observe the long term interactions among the variables. With multiple periods of data, they make the results of research more complete and objective.

## Pre-publication history

The pre-publication history for this paper can be accessed here:


